# The Gospel of Personal Service

**Published:** 1915-06-12

**Authors:** 


### THE GOSPEL OF PERSONAL SERVICE.

Hospital Sunday is the classic example of an anniversary day specially set apart for the benefit of charity. Not only so, but in this year of war it is the outstanding example of an annual charitable function which there has been no suggestion to discard. The street collections in different localities, the charitable bazaars and garden-parties, the hospital festival dinner itself, have all been waived. The annual appeal to meet a deficiency, the special appeal to provide funds for some new extension of work, have mostly been scratched, like racing, as unsuited to the sentiment and stern necessity of the year. But Hospital Sunday is an exception. This one great day of appeal on behalf of the voluntary hospitals will be honoured for the fifty-sixth year in succession from every pulpit, even from those in which aspects of the war have threatened to oust the traditional messages of other festivals, Sundays, and red-letter days. Nor does this apparent exception to the fear that the claims of charity are not likely to be attended to in time of war require much explanation. Hospital Sunday is, in fact, the festival which symbolises the great idea which is beginning to electrify England—that idea which is the tradition of the voluntary hospitals, of personal service. The need for this has been stirring uneasily in the minds of Englishmen during the past nine months. The newspapers have fixed on an outward expression of it in the consideration which they have given to compulsory military service, and to the need which is also being urged for what is called the mobilisation of all our resources. This is the underlying reason why Hospital Sunday, alone among the festivals of charity, maintains its position this year when so many smaller examples have yielded to the circumstance of war. But, indeed, besides this, the ray of light and cheerfulness, the reconstructive activity which has been thrown into high relief amid all the ruin of destruction, has been the work which the voluntary hospitals have been doing for the wounded and the sick, the poisoned and disabled of the war. It is a great thought that the gospel of personal service which the voluntary system of hospital support has preached on Hospital Sunday through an almost undisturbed half-century of peace should have become a gospel for the veriest commercial newspaper reader in the present time of war. Small wonder, then, that the persistent, quiet voice of voluntary hospital teaching is become a clarion call, though, alas! it has required a European conflagration to illustrate the necessity for it.

Nowadays, then, the war rather than the voluntary hospital is compelling men's attention to the fact that personal service must be the mainspring of any man in every nation who values his life and its mission, as a high calling for which the existence of the individual citizen is only the period of office of a trustee. The gospel is on every man's lips; the work which the hospitals are now performing is before every man's eyes. The work which these institutions do, and the need for it, is more clearly realised by the general public than ever before. Its value is beyond dispute. On this occasion Hospital Sunday, therefore, has not so much to drive the lesson home as to remind the nation that an immense sum of money is necessary for the work of the voluntary hospitals to be carried on under the heavy imposition which is now being laid upon them. For once their example overshadows in the public eye the teaching which they exist so largely to inculcate; and so the public has merely to be reminded that the labourer is worthy of his hire. It would, indeed, be a strange and lamentable thing if this year's work, which has surpassed all previous efforts, should not result in a record collection on Hospital Sunday also. Imagine a vast public hall filled to overflowing with a crowd of people who have assembled to assist, by founding a great fund, the cause of the widows and dependants of those fallen in the war, full of enthusiasm, cheering the speakers, applauding the resolutions—imagine this vast assembly so excited by good intentions as to break up having forgotten to call for any subscriptions or to pass round the hat. Such a situation is easily conceivable, and has probably happened before now, but the abysmal folly of the proceeding is exactly similar to what will happen this year if the total subscribed to the Hospital Sunday Fund does not beat all previous records. The public knows the facts about the hospitals, is full of enthusiasm on their behalf, is genuinely moved by the work which they are performing. But this knowledge, enthusiasm, and gratitude must be crystallised into a practical form, so that the good work may be maintained from day to day. Hospital Sunday exists so to crystallise it.

Our voluntary hospitals depend entirely on what has been, and still is, given voluntarily to them by way of donation, annual subscription, or legacy. It is true that the War Office is giving a sum round about 4s. a day to many, though not all, of the hospitals for every wounded soldier they receive. But this sum does not cover all expenses, quite apart from the fact that hospitals have their duty to civil patients as well. By building hut wards and emergency structures of various kinds many institutions are still able to take in as many ordinary patients as before. All the hospitals continue to receive a large proportion of them, and the awakening of the national conscience which has led to the above offer of a Government grant for wounded soldiers must penetrate every civilian also, and lead him "to do his bit," as we say, by subscribing to the institutions in whose good keeping the lives of so many of our friends, sons, brothers, and husbands are confided to-day.

The part which the clergy have taken in bringing home to the people the need for prosecuting the war and the necessity for personal service has been one of the most remarkable features of the past few months. Almost every pulpit has echoed the call to arms. Every pulpit certainly has preached the gospel of personal service. Let the clergy, then, be reminded with gratitude and pride that Hospital Sunday was founded by one of their own order, Canon Miller, in 1859. Its gospel of personal service is their gospel, its protagonist one of themselves. Indeed, it may be said with truth that Canon Miller founded Hospital Sunday, not only as a means for organising the Central collection on behalf of the voluntary hospitals, but because the great thought came to him that the voluntary system itself was but the doctrine of personal service organised and put into practice with reference to the healing of disease. Hospitals, of course, have paid officials; but they depend almost exclusively for their management and control on the brains and time of men and women who voluntarily give their service on committees, as chairmen, treasurers, in ladies' guilds, and subsidiary organisations of all sorts. The medical staffs, as we all know, are not, as a whole, paid anything for their services, but take their material reward in the experience they gain and the prestige which is inherent in their positions. The paid officials are brought up equally in the same tradition. According to commercial standards, beyond a doubt, they are much underpaid, and gain their reward in the satisfaction which comes to those who, means for bed and board once granted, devote themselves to superselfish work. Thus, in short, hospital work is personal service given by all, paid and unpaid, who engage in it, and Hospital Sunday, on which this gospel of service is preached, may be regarded as the festival of Churchmanship actually at work in the world. Founded by the clergy, carried on by them, and this year with the message it has taught embraced by the whole nation, what preacher will not thrill with pride as he repeats the claim of this glorious gospel, and what congregation will be so dull to its meaning as not to give of their best, that the sum total of hard cash subscribed may be as much a "record" this year as the work of the hospitals has been?

